# Development of Triamcinolone Acetonide-Loaded Microemulsion as a Prospective Ophthalmic Delivery System for Treatment of Uveitis: In Vitro and In Vivo Evaluation

**DOI:** 10.3390/pharmaceutics13040444

**Published:** 2021-03-25

**Authors:** Alaa Mahran, Sayed Ismail, Ayat A. Allam

**Affiliations:** 1 Department of Pharmaceutics, Faculty of Pharmacy, Assiut University, Assiut 71526, Egypt; AlaaAhmed@pharm.aun.edu.eg (A.M.); sayed.hussien@pharm.aun.edu.eg (S.I.); 2 Department of Pharmaceutics, Faculty of Pharmacy, Sphinx University, New Assiut City, Assiut 71515, Egypt; 3 Assiut International Center of Nanomedicine, Al-Rajhy Liver Hospital, Assiut University, Assiut 71515, Egypt

**Keywords:** uveitis rabbit model, histopathological examination, Cremophor EL, ocular delivery, ocular inflammation

## Abstract

Treatment of uveitis (i.e., inflammation of the uvea) is challenging due to lack of convenient ophthalmic dosage forms. This work is aimed to determine the efficiency of triamcinolone acetonide (TA)-loaded microemulsion as an ophthalmic delivery system for the treatment of uveitis. Water titration method was used to construct different pseudo-ternary phase diagrams. Twelve microemulsion formulations were prepared using oleic acid, Cremophor EL, and propylene glycol. Among all tested formulations, Formulation F3, composed of oil: surfactant-co-surfactant (1:1): water (15:35:50% *w/w*, respectively), was found to be stable and showed acceptable pH, viscosity, conductivity, droplet size (211 ± 1.4 nm), and zeta potential (−25 ± 1.7 mV) and almost complete in vitro drug release within 24 h. The in vivo performance of the optimized formulation was evaluated in experimentally uveitis-induced rabbit model and compared with a commercial TA suspension (i.e., Kenacort^®^-A) either topically or by subconjunctival injection. Ocular inflammation was evaluated by clinical examination, white blood cell count, protein content measurement, and histopathological examination. The developed TA-loaded microemulsion showed superior therapeutic efficiency in the treatment of uveitis with high patient compliance compared to commercial suspension. Hence, it could be considered as a potential ocular treatment option in controlling of uveitis.

## 1. Introduction

Uveitis is an inflammation in the uveal layer of the eye that lies below the sclera and cornea which includes iris, choroid, and ciliary body. It may also affect retina, vitreous, sclera, and optic nerve. It is associated with about 10% of the blindness cases in the whole world [1]. Uveitis may be classified anatomically into anterior uveitis which includes anterior chamber, intermediate uveitis involves vitreous body, posterior uveitis which affects choroid and retina, and panuveitis which affects all layers of the uvea. Most challenges in dealing with uveitis are related to the treatment of the posterior segment inflammation. Corticosteroids (e.g., dexamethasone, triamcinolone acetonide (TA), and fluocinolone acetonide) and immunosuppressant agents are the commonly used agents in the treatment of uveitis [2].

TA is a synthetic corticosteroid that is widely used in the treatment of several inflammatory conditions. Regarding its potency, it is more potent than triamcinolone and about eight times more potent than prednisolone. It is effective in the treatment of several ocular conditions (e.g., diabetic macular edema, refractory cystoid macular edema, and uveitis) and other ocular inflammatory conditions that require long-term steroid administration [3]. Topical delivery of TA to the posterior part of the eye is problematic due to presence of strong defensive anatomical, physiological, and biochemical barriers of the posterior eye structure, thus the drug is injected directly to the eye either with intravitreal or periocular route [4]. These targeted methods increase TA delivery to the deep ocular structures and vitreous cavity and also provide prolonged duration of action because TA is insoluble in body fluids and provides a depot effect [5]. Although the intravitreal IVT injection of corticosteroids is an efficient method, it is associated with several side effects which may be injection-related (e.g., infectious endophthalmitis, pseudo-endophthalmitis, and retinal detachment) or steroid-related (e.g., intraocular pressure elevation (IOP) and cataract formation) [6]. Although periocular injection is less invasive and less painful than IVT, it results in a small amount of the drug in the vitreous cavity and, thus, it fails to achieve therapeutic drug concentrations. Therefore, higher TA doses are required that could increase IOP. Additionally, periocular injection is associated with conjunctival ulceration, infectious scleritis, blepharoptosis, and conjunctival ischemia [7]. 

Consequently, less invasive methods for TA administration that can achieve enough drug contact time with ocular surface and attain therapeutic drug concentrations at the target site are greatly required. Various approaches have been developed to increase ophthalmic delivery of TA using topical administration route. Nanotechnology-based ocular delivery systems enable efficient and safe drug delivery to its target site [8]. These approaches include: liposomes [9], nanoparticles [10], nanostructured lipid carriers [11], and microemulsions [12].

Microemulsion is a clear, isotropic, thermodynamically stable system that is formed in a spontaneous manner without any significant energy input, by mixing the oily phase with the aqueous phase in the presence of high amphiphilic concentration (either surfactant alone or in combination with co-surfactant), which decreases the interfacial tension to a very low value and leads to the formation of one dispersion into the other. It is composed of oil, water, surfactant, and co-surfactant in appropriate ratios. Microemulsion has three different types: oil in water (O/W), water in oil (W/O), and bicontinuous structure, so it can be used as a delivery system for both hydrophilic and hydrophobic drugs [13]. Various studies demonstrated the efficiency of the microemulsion as an ocular drug delivery system for delivering many drugs to different ocular segments [14] such as timolol [15], pilocarpine nitrate [16], dexamethasone [17], and chloramphenicol [18]. The high surfactant and co-surfactant concentration in the microemulsion may lead to an increase in its cellular uptake and membrane penetrability by loosening the tight junction between the epithelial cells, leading to the disruption of the integrity of the membrane and hence, this may increase the drug delivery to the posterior structure of the eye [19]. The microemulsion components were initially selected on the basis of their ophthalmic safety. Vegetable oils such as castor oil, olive oil, corn oil, and soya bean oil [20,21] and monounsaturated fatty acids (e.g., oleic acid) [22] are the most widely used oily phase in microemulsion preparation and were chosen due to their safety for application to the eye. Cremophor EL [17], Tween 80 [23], and Brij 35 [24] were screened as surfactants, while polyethylene glycol 400 (PEG 400) [12], propylene glycol (PG) [25], and glycerol [21] were screened as co-surfactants. The high hydrophilic lipophilic balance (HLB) values of all tested nonionic surfactants (i.e., >10) are responsible for the formation of O/W type of microemulsion, which is more preferred due to its stability upon dilution by biological fluid. Thus, the aim of this work was to formulate and evaluate both in vitro/in vivo performance of TA-loaded microemulsion as a topical ophthalmic delivery system for the treatment of intermediate or posterior uveitis in a rabbit animal model. 

## 2. Materials and Methods

### 2.1. Materials

TA was a gift sample from PHARCO Pharmaceuticals Inc. (Alexandria, Egypt). Oleic acid, castor oil, olive oil, corn oil, and soya bean oil were purchased from Alpha CHEM Co. (Cairo, Egypt). Cremophor EL (polyoxy 35 castor oil) was a gift sample from BASF (Ludwigshafen, Germany). Tween 80 was obtained from El-Naser Pharmaceutical Chemicals Co. (Cairo, Egypt). Brij 35 was purchased from E. Merck (Darmstadt, Germany). PEG 400, PG, and glycerol were purchased from Iso-Chem Co. (Cairo, Egypt). Bovine serum albumin (BSA) was obtained from (Sigma-Aldrich, St. Louis, MO, USA).

### 2.2. Solubility Study

TA solubility was determined through addition of a known excess amount of the drug in glass vials each containing 2 mL of a tested vehicle (i.e., oleic acid, castor oil, olive oil, corn oil, soyabean oil, tween 80, Cremophor EL, Brij 35, PEG 400, PG, glycerol). The mixtures were placed in a thermostatically controlled shaking water bath (37 ± 0.5 °C) at 150 rpm for 72 h to achieve equilibrium. Then, samples were centrifuged at 6000 rpm for 15 min [25]. The concentration of TA in the supernatants was determined using a UV-vis spectrophotometer at *ʎ_max_* of 239 nm. All experiments were conducted in triplicates and TA solubility (µg/mL) in each vehicle was recorded as mean value ± SD.

### 2.3. Pseudo-Ternary Phase Diagram Study

Water titration method was used to construct pseudo-ternary phase diagrams at room temperature (25 °C) [26]. Because microemulsions are made of four components, the total surfactant to co-surfactant ratio (S_mix_) can be represented by one axis of the pseudo-ternary phase diagram and the remaining two axes represent oil and water. In this study, castor oil and oleic acid were used as oily phases, Tween 80, Cremophor EL, and Brij 35 as surfactants and PEG 400, PG, and glycerol as co-surfactants and distilled water as the aqueous phase. Different pseudo-ternary phase diagrams were constructed according to Table 1. 

Each phase diagram was developed by mixing the specific weight ratio of surfactant and co-surfactant (S_mix_) with oil in different ratios (1:9, 2:8, 3:7, 4:6, 5:5, 6:4, 7:3, 8:2, and 9:1% *w/w*). Water was then added (known volume) under moderate magnetic stirring in order to attain water content between 5% and 95% of the total formulation weight. After being equilibrated overnight, visual observation of the prepared formulations was made to determine whether they were microemulsions (i.e., translucent with light yellow opalescence) or simple emulsions (i.e., appearance of white turbidity). To distinguish between W/O or O/W microemulsion and bicontinuous microemulsion, the bicontinuous systems were claimed for those clear and highly viscous mixtures that did not show a change in the meniscus after tilted to an angle 90° [27]. The excipient ratios that have resulted in the formation of microemulsions were blotted as points on the pseudo-ternary phase diagrams using CHEMIX School program version 5© (Arne Standnes, Bergen, Norway). The microemulsion area of the pseudo-ternary diagrams was determined using weight and cut method [28].

### 2.4. Formulation of TA-Loaded Microemulsions

The selected formulations were obtained from pseudo-ternary phase diagrams constructed using oleic acid as an oily phase, Cremophor EL as a surfactant, and PG as a co-surfactant in three different S_mix_ weight ratios (1:1, 1:2, and 2:1). Four formulations were chosen from the microemulsion region as expressed in Table 2. Each formulation was loaded with 0.05% *w/w* TA. The selection was initially based on the amount of the oil, which should be ≤20% *w/w* to decrease the greasy effect of the oil, which may result in patient discomfort. The amount of the surfactant and co-surfactant (S_mix_) in each formulation should be ≤50% *w/w* to minimize their corresponding irritation effect [29]. In order to prepare drug-loaded microemulsion formulations, the oil was added first to the S_mix_ mixture and stirred gently until complete mixing. Then, 0.05% *w/w* TA was added with stirring until it had been perfectly dissolved. Then, a defined volume of phosphate buffer solution (pH 7.4, 0.0667 M) was titrated slowly with gentle stirring until microemulsion was obtained. The prepared microemulsions were finally stored in closed sterile glass vials. 

### 2.5. Thermodynamic Stability Study

First, the selected formulations were subjected to centrifugation at 5000 rpm for 30 min. Then, the passed formulations were subjected to six heating (40 °C) and cooling (4 °C) cycles for at least 48 h at each temperature. Finally, those stable formulations were exposed to three freeze-thaw cycles at −21 °C and 25 °C for 48 h at each temperature [30]. Only passed microemulsion formulations were chosen for further evaluation.

### 2.6. Microemulsion Characterization

The pH of the selected microemulsion formulations was measured at 25 °C using Jenway 3505 (Jenway Ltd., Feslted, Dunmow, Essex, United Kingdom) [31].

The viscosities of the undiluted preparations were determined at 25 °C using Brookfield Programmable Rheometer (Model RVDV-III U), Brookfield Engineering laboratories, Inc., Stoughton, MA, USA) with # 94 (ULA) spindle at 150 rpm [32]. 

Measurement of the electrical conductivity was carried out at 25 °C by means of a pH/mV/ISE Temperature Bench meter (Hanna HI 4222, Padova, Italy). The electrode was placed in the microemulsion preparation until equilibrium was attained and reading became stable [23].

Droplet size, polydispersity index (PDI), and zeta potential of the selected microemulsion formulations were measured by dynamic light scattering (DLS) technique using Malvern Zetasizer Nano Series ZS 90 (Malvern Instruments, Malvern, Worcestershire, UK) after 1000-fold dilution with distilled water [33]. All measurements were carried out three times and recorded as mean value ± SD.

### 2.7. In Vitro Release Experiment

Release of the drug from different formulations and dissolution of the free drug powder were evaluated using dialysis bag method (molecular weight cut-off 12,000–14,000 Da) [32]. In the donor compartment, 2 g of the microemulsion formulation (equivalent to 1 mg TA) was placed. The release medium, which consisted of 30 mL of simulated tear fluid (STF) solution (pH 7.4) [34] and 1.5% *w/v* sodium lauryl sulphate, was placed in the receptor compartment. The procedure was carried out in a thermostatically controlled shaking water bath operating at 50 rpm and at a temperature of 34 ± 0.5 °C [35]. At defined time intervals, 3 mL of the release medium was withdrawn from the receptor compartment and replaced with a fresh release medium to preserve sink conditions [21]. All samples were assayed using spectrophotometer at *λ_max_* 239 nm. The release experiments were performed in triplicates and the mean % of cumulative drug release ± SD were reported.

To study drug release kinetics, in vitro release data were fitted in different kinetic models including zero order, first order and Higuchi diffusion model. The data were also fitted to Korsmeyer–Peppas equation to determine the drug diffusion mechanism by analyzing the diffusion exponent (*n*). If *n* ≤ 0.49, the release follows Fickian mechanism, if 0.5 ≤ *n* ≤ 0.8, the release follows a non-Fickian mechanism [36].

### 2.8. Further Characterization of the Selected Formulation

#### 2.8.1. Rheological Behavior

The flowability of the selected formulation (i.e., F3) was studied at different angular velocities ranging from 10 rpm to 200 rpm, then angular velocity was decreased from 200 to 10 rpm, retaining a period of 60 s at each rpm [37]. The viscosity was determined using the average of two readings. The experiments were carried out in triplicates and mean values ± SD were recorded.

#### 2.8.2. Transmission Electron Microscope

The morphology of TA-loaded F3 microemulsion was examined using transmission electron microscope (TEM) TECNAI G2 Spirit Twin (FEI, USA). The sample was 1000-fold diluted with distilled water, then one drop was added on copper grids, and permitted to stand for 5 min. One drop of uranyl acetate (2% *w/v*) solution was used to stain the grids [38].

#### 2.8.3. Stability Study

The stability of the selected microemulsion formulation F3 was evaluated over 6 months at 4 °C and 25 °C using the following parameters: mean droplet size, PDI, zeta potential, pH, and appearance. The parameters were evaluated by the same methods adopted before for the samples.

### 2.9. In Vivo Study

#### 2.9.1. Animals

The research protocol of animal studies was reviewed and approved by the Institutional Animal Ethical Committee of the Faculty of Medicine, Assiut University, Egypt, and adhered to the Guide for the Care and Use of Laboratory Animals, Eighth Edition, National Academies Press, Washington, DC, USA (ethical approval number S7-19 on 1 October 2019). Male domestic rabbits weighing 1.5–2 Kg and do not have any abnormalities or damage of the ocular surface were chosen. They were obtained from the animal house of Faculty of Medicine, Assiut University. Animals were housed under 12 h dark-light cycle at 25 °C and allowed water and standard laboratory chow ad libitum. 

#### 2.9.2. Ocular Irritation Test

Ocular tolerability of the selected microemulsion formulation F3 was evaluated according to modified Draize test using a penlight in 3 male domestic rabbits [17]. First, the selected formulation F3 without the drug was prepared using isotonic Sorenson phosphate buffer and sterilized using autoclave. Then, 50 µL of this formulation was applied for 1 week twice per day into the conjunctival sac of the right eye of 3 rabbits. Similarly, the left eye received isotonic normal saline and act as negative control. Then, the possible damage caused to the cornea, iris, and conjunctiva was evaluated by visual inspection using a scale of weighted scores. The corneal opacity (i.e., area most dense taken for reading) was scored from 0 to 4, iris (severity of iritis) was scored from 0 to 2, and the conjunctival redness (palpebral and bulbar conjunctivae) was scored from 0 to 3 [39]. The total ocular irritation scores were calculated by summation the individual irritation scores of the cornea, iris, and conjunctiva. The ocular reaction, which indicates ocular irritation, may be defined as a result meeting or exceeding specific numerical cut-offs, such as corneal opacity score ≥ 1, iritis score ≥ 1, or conjunctival score ≥ 2. After 1 week, animals used in this test were sacrificed and eyes were isolated and preserved in 10% neutral-buffered formalin for further histopathological examination.

#### 2.9.3. Induction of Uveitis

Uveitis was induced in 12 rabbits according to the previously reported procedure [40]: briefly, at the beginning, the rabbits were anesthetized with ketamine (25 mg/Kg) (Inresa Arzneinmittel GmbH Co., Freiburg, Germany) and midazolam (1 mg/Kg) (Amoun Pharmaceuticals Co., Egypt). Then, lidocaine solution was applied topically to anesthetize the ocular surface of the right eye of each rabbit. After anesthesia, 0.2 mL of normal saline containing 40 mg of bovine serum albumin (BSA) was intravitreally injected at the 12 o’clock position, 3–4 mm posterior to the limbus through part in ciliary body called pars plana with a 30-gauge needle. Similarly, the left eyes were injected with 0.2 mL of isotonic normal saline to serve as a negative control. At the end of the procedure, tobramycin ophthalmic solution was applied topically after injection. Twenty-four hours after induction, the right eyes of individual rabbits were examined for induction of uveitis. 

Then, the potential therapeutic efficiency of the selected microemulsion formulation (F3) for uveitis treatment was compared to the commercially available TA suspension (Kenacort^®^-A 40, GlaxoSmithKline, London, UK). TA suspension was administrated not only as a subconjunctival injection but also as a suspension instilled topically into the eye. The rabbits were randomly divided into 4 groups, each consisting of 3 rabbits. The first group remained untreated and received normal saline topically and acted as a positive control. The second and third groups were treated twice per day for 1 week topically with 60 µL of the selected formulation F3 (0.05% *w/w* TA) and 70 µL of TA suspension (0.04% *w/v*), respectively. The fourth group received one subconjunctival injection (100 µL) of TA suspension (equivalent to 0.4 mg TA, 0.4% *w/v*).

The effect of F3 on uveitis induced in rabbits was assessed by scoring of uveitis clinical signs, anterior chamber white blood cells (WBCs) count and protein content measurement, and finally by histopathological studies.

##### Scoring or Clinical Observation of Uveitis

Rabbits’ eyes were examined daily for signs of uveitis including conjunctival congestion, iris vessel congestion, corneal clarity. Inflammatory responses are graded and summarized in Table 3.

##### Anterior Chamber White Blood Cells (WBCs) Count and Protein Content

The intensity of inflammation and integrity of blood aqueous barrier (BAB) in the treated groups were also assessed indirectly by counting the number of WBCs and determining the content of protein that infiltrated the anterior chamber within the tested eyes. 

For these studies, 100 µL aqueous humor was collected from each rabbit in the four groups through an anterior chamber puncture using a 30-gauge needle in three different occasions: normal healthy rabbit eyes, rabbit eyes with induced uveitis (24 h after BSA injection), and finally after seven days treatment. Immediately after sample collection, 100 µL of 0.1% Ethylenediaminetertaacetic acid EDTA solution was added to them. The number of the WBCs was calculated using a hemocytometer under light microscopy. In addition, the total protein concentration was measured by using pyrogallol red protein assay reagent kit (Sigma-Aldrich, St. Louis, United States) at *ʎ_max_* 598 nm [42]. 

The percent reduction in WBCs count and protein content was calculated by the following equation [43]:

(1) $$\%\;\mathrm{Reduction}\;\mathrm{in}\;\mathrm{WBCs}\;\mathrm{and}\;\mathrm{protein}\;\mathrm{content}\;=\;\frac{D_{24\;h}-D_{7\mathrm{th}\;\mathrm{day}}}{D_{24\;h}}$$

where (*D*_24h_) is data of the anterior chamber samples obtained after 24 h from uveitis induction and (*D*_7th day_) is data obtained after seven days of treatment.

##### Histopathology

After seven days of treatment, the rabbits were euthanized. The whole right and left eyes were dissected from all groups and fixed in 10% neutral-buffered formalin for 48 h. Then, these eyes were sliced just behind the cornea to divide the ball into anterior and posterior parts. Selected samples represented the cornea, sclera, ciliary body and their processes, iris, retina, and choroid were fixed in 10% neutral buffered formalin for 24 h, processed, sectioned at 4–6 µm thickness, stained by Hematoxylin and Eosin (H and E), and finally, examined by light microscope (Olympus microscope, CX3I, Tokyo, Japan) and photographed using a digital camera (Olympus, Camedia C-5060, Tokyo, Japan). Tissues were scored from 0 (normal) to 4 (marked) signs of inflammation. Scores were combined to give a total inflammatory score of 20 using the following criteria: (i) edema or congestion in the cornea, iris, ciliary process, and choroid, (ii) inflammatory cell infiltration in the cornea, ciliary process, and retinal tissues, and (iii) neovascularization in the cornea [44]. 

### 2.10. Statistical Analysis

Statistical analyses were carried out using GraphPad Prism version 7.01 for windows, GraphPad Software, La Jolla, CA, USA. One-way ANOVA followed by Tukey post hoc test were used to analyze the differences between experimental groups. Student’s *t*-test was employed for pairwise comparison. The differences were considered significant at *p* ≤ 0.05. 

## 3. Results and Discussion

### 3.1. Drug Analysis

Figure S1 represents UV scanning of TA solution in methanol. TA has a maximum absorbance (*λ_max_*) at 239 nm. Figures S2 and S3 represent the calibration curve of TA in methanol and simulated tear fluid, respectively. 

### 3.2. Solubility Study

Drug solubility in an appropriate amount of the microemulsion excipients is an important consideration for successful formulation to maintain the drug in the solubilized form [45]. As shown in Table 4, castor oil showed a significant solubilizing capability of TA compared to other tested oils (*p* < 0.05) followed by oleic acid. The exceptional high drug solubility in castor oil compared with other vegetable oils can be attributed to the presence of ricinoleic acid which has a hydroxyl functional group that increases castor oil polarity compared to other oils [46]. These results coincide with those found by Padula et al. [47], who reported low solubility of TA in various vegetable oils.

TA solubility in surfactants was considerably much greater than that of oils and co-surfactants (*p* < 0.05), which can be correlated to its intermediate partition coefficient (log *p* 2.53) [48]. Brij 35 showed a significant solubilizing capability for TA compared to other surfactants (*p* < 0.05). The hydrophilic nature of the used co-surfactants (i.e., PEG 400, PG, and glycerol) may be the reason for their corresponding lower solubilizing capacity for the drug. Compared to other co-surfactants, PEG 400 exhibited the highest solubilizing capacity for the drug. However, the final selection among the surfactants, co-surfactants, as well as castor oil and oleic acid will be further assured depending on emulsification efficiency [49].

### 3.3. Pseudo-Ternary Phase Diagram Study

Pseudo-ternary phase diagram is an efficient method to study the phase behavior of a mixture and the effect of its composition on the microemulsion area [50]. The shaded area within the diagram represents the transparent microemulsion region containing various percentages of constituents, while the remaining area represents the turbid and simple emulsions based on visual observation. 

#### 3.3.1. The Effect of Oil Composition on the Microemulsion Area

Chemical structure of the oily phase influences the efficiency of microemulsion formation. Oil components (i.e., fatty acids) can penetrate deeply and cause swelling of the tail region of the surfactant monolayer and affect its curvature. The hydrophobic chain length of the oil phase and its volume have greater effect on this penetration. It was hypothesized that oils with an excessively long hydrocarbon chain, and hence bulk structure, result in a small microemulsion area [51].

As shown in Figures S1 and S2 (Supplementary Data), oleic acid resulted in larger microemulsion areas with all tested surfactants compared with castor oil. These results can be attributed to the simple structure of oleic acid compared to castor oil, enabling deep penetration of the oil to the tail region of the surfactant and causing swelling of this region to a greater extent than did castor oil, which is a vegetable oil with bulk structure [52]. 

In addition to its corresponding large microemulsion area, oleic acid demonstrates a penetration enhancing activity for several drugs administered through various routes by increasing the fluidity of the lipid bilayer and enhance drug penetration through different physiological barriers such as skin, cornea, brain, and mucous membrane [22]. For these reasons, oleic acid was chosen as the oily phase for microemulsion formulation and subjected to further studies.

#### 3.3.2. The Effect of the Surfactant Structure on the Microemulsion Area

It is obvious from Figures S1 and S2 (Supplementary Data) that the largest microemulsion areas were obtained when Cremophor EL was used as a surfactant with both castor oil and oleic acid. Although Tween 80 formed stable microemulsions at high water content with oleic acid, it failed to form a stable microemulsion with castor oil. It was also observed that Brij 35 did not form stable microemulsions at high water content, thus it is unsuitable for O/W microemulsion formation. 

Regarding surfactant structure, the presence of fluidizing groups such as double bonds or branching in the hydrophobic chain of the surfactant promoted the formation of the microemulsion and increased the microemulsion area in the pseudo-ternary diagram because these groups render the surfactant more flexible to adopt different curvature [53]. Further, they may increase the penetration of the alkyl chain of the surfactant into the oily phase and increase the oil uptake in the surfactant monolayer [54]. 

In comparison with Tween 80 and Brij 35, Cremophor EL has a branched alkyl chain structure which makes oil–water interface more flexible and improves the penetration of oil into the surfactant film [55,56]. As a conclusion of the previous results, Cremophor EL was chosen as a surfactant of choice and subjected for further studies. 

#### 3.3.3. The Effect of Co-Surfactant on the Microemulsion Area and Phase Transition

Generally, physicochemical characteristics of the co-surfactants may affect the properties of surfactants in aqueous solutions by reducing the interfacial tension or may affect surfactant packing and increase the flexibility of the surfactant film to take up adequate curvatures necessary for microemulsion formation over a wide range of composition [57]. In this study, three co-surfactants (i.e., PEG 400, Glycerol, and PG) in three different S_mix_ ratios (i.e., 1:1, 1:2, and 2:1 *w/w*, respectively) were chosen to figure out the effect of their structures and S_mix_ ratios on the microemulsion area and on the fluidity of the interfacial film.

**Figure 1:**
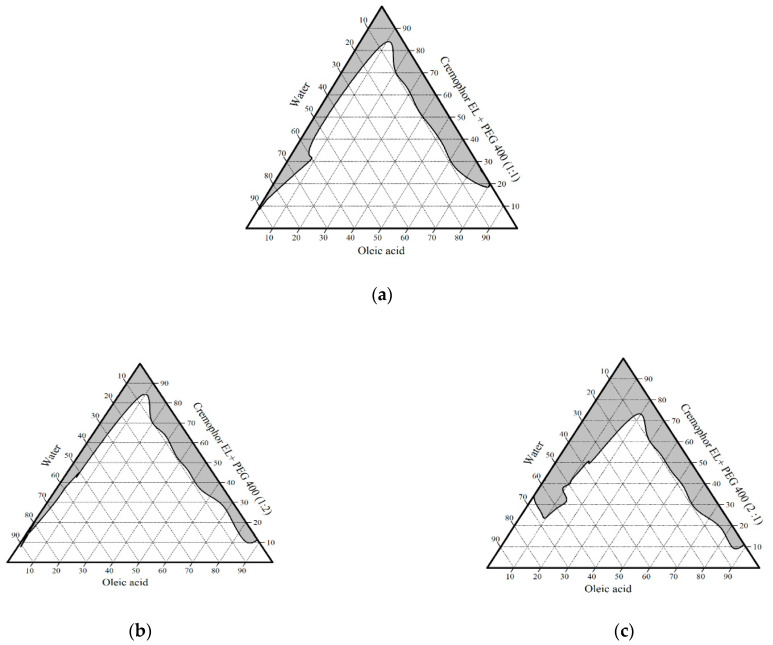
Pseudo-ternary phase diagrams of group III containing oleic acid as oil phase, Cremophor EL as a surfactant, and polyethylene glycol 400 (PEG 400) as a co-surfactant in ratio S:C (**a**)1:1, (**b**) 1:2, and (**c**) 2:1.

**Figure 2:**
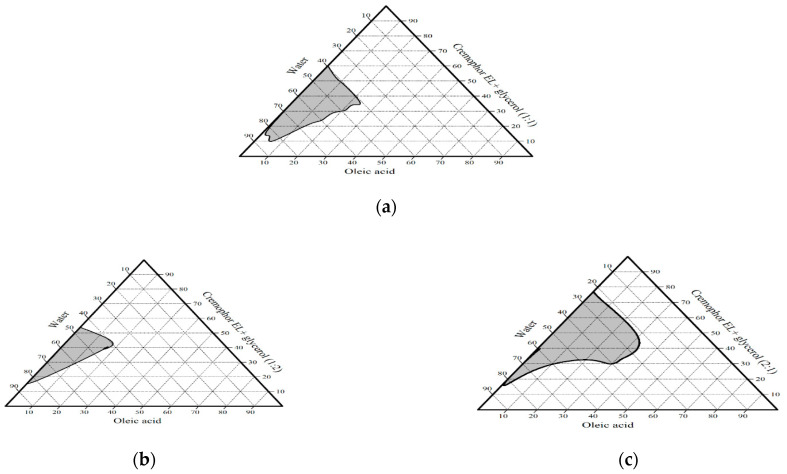
Pseudo-ternary phase diagrams of group IV containing oleic acid as oil phase, Cremophor EL as a surfactant, and glycerol as a co-surfactant in ratio S:C (**a**) 1:1, (**b**) 1:2, and (**c**) 2:1.

**Figure 3:**
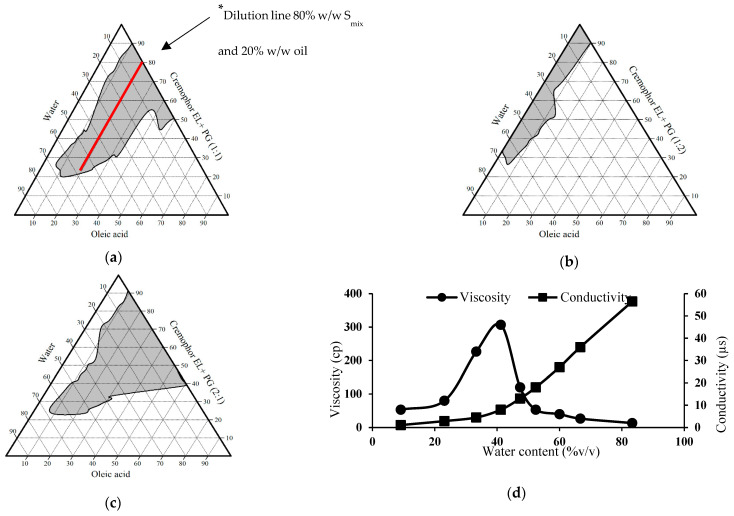
Pseudo-ternary phase diagrams of group V containing oleic acid as oil phase, Cremophor EL as a surfactant, and propylene glycol (PG) as a co-surfactant in ratio S:C (**a**) 1:1, (**b**) 1:2, and **(c**) 2:1 and (**d**); viscosity and conductivity measurements to study the effect of water content on microemulsion phase transition. (*) Shows the dilution line of the initial mixtures used in viscosity and conductivity assessment.

From Figure 1, Figure 2 and Figure 3 as well as Table 5, it is obvious that the largest microemulsion area was obtained when PG was used as a co-surfactant. Additionally, at high oil content, only PEG 400 had the ability to form W/O microemulsion in comparison with PG, which gives W/O microemulsion only at intermediate oil content, while glycerol failed to produce W/O type of microemulsion. The effect of co-surfactant structure on the formed type of microemulsion can be explained by decreasing the ability of co-surfactant to penetrate the interfacial film of the surfactant by increasing its polarity, so it mostly locates in the aqueous phase and prefers formation of O/W type. PG and glycerol with both short-chain alcohols (i.e., having two and three hydroxyl groups) with log *p* values, −0.92 and −1.76, respectively, are more polar than PEG 400 with log *p* −0.38, so they prefer the interaction with the hydrophilic head group of the surfactant than the tail. This interaction cause swelling of the head region and prefer formation of O/W microemulsion than W/O type. Nevertheless, the difference in the microemulsion area between PG and glycerol may be due to their hydroxyl groups number, as by increasing the number of hydroxyl groups of the co-surfactant, the microemulsion area is reduced [58]. On the other hand, PEG 400 is a polyether co-surfactant, which prefers penetration of the interfacial film and swelling of the hydrophobic chains of the surfactant molecules more than the head groups and hence promotes the formation of W/O microemulsion [59]. 

In addition to the percent of microemulsion area in the pseudo-ternary diagram, Table 5 also lists the maximum amount of oil solubilized by various systems studied. It was observed that when the relative concentration of co-surfactant decreased, the maximum oil incorporated in the O/W microemulsions increased, leading to increasing the microemulsion area. High co-surfactant to surfactant ratio results in destabilization of the microemulsion and hence reduction of the microemulsion area [60,61].

Additionally, the rigidity and flexibility of the interfacial film was also affected by co-surfactant concentration. Table 5 illustrates the effect of co-surfactant on the microemulsion phase transition and compares the percent of fluid region (A_fluid_) which comprises O/W and W/O microemulsion with the percent of bicontinuous region (A_bi_). It was noted that increasing surfactant to co-surfactant weight ratio resulted in increasing the area of the bicontinuous region and hence decreasing the fluid region. The co-surfactant showed greater effect on the microemulsion phase transition from W/O to bicontinuous and finally to O/W microemulsion. Microemulsion systems containing PEG 400 and glycerol as co-surfactants, in all surfactant to co-surfactant ratios, undergo fluid-gel-fluid transition. When PG was used as a co-surfactant, at surfactant to co-surfactant ratios, 2:1 and 1:1, respectively, the microemulsion systems showed similar fluid-gel-fluid transition; however, at a ratio of 1:2, the bicontinuous region was abolished from the phase diagram and the microemulsion region became entirely fluid in nature. These results may be attributed to the ability of the high co-surfactant to surfactant ratio to decrease the interfacial tension and increase the fluidity of the interface and hence decrease bicontinuous region and increase W/O and O/W microemulsion region (fluid region) [28]. From the previous results, PG was selected as a co-surfactant of choice based on its corresponding large microemulsion areas with Cremophor EL and oleic acid in all tested ratios. 

#### 3.3.4. The Effect of Water Volume on the Phase Transition

There is a strong correlation between phase transition and increasing the volume fraction of the aqueous phase (*Φ*w) in microemulsion systems [59]. In order to figure out these microstructural changes, viscosity and electrical conductivity were measured because they largely depend on the concentration and interaction of the dispersed phase [62]. First samples were obtained from Figure 3a by water titration of initial mixtures containing 80% S_mix_ (Cremophor EL and PG in a weight ratio 1:1) and 20% oleic acid. Then the obtained samples were subjected to viscosity and electrical conductivity studies. Figure 3d illustrates the variation of the viscosity and the electro-conductive behavior of the prepared samples as a function of water volume. At low water content (i.e., <20%), the formed microemulsions appeared to have low viscosity and the conductivity value was almost zero, which may indicate the formation of W/O microemulsion with water droplets dispersed within the oil phase based on the reported data of having the water-continuous microemulsion higher electrical conductivity than the oil-continuous one [63]. Then, by increasing water content between 20–50%, there was a sharp increase in the viscosity and slight increase in the electrical conductivity (linear relationship), which may be due to the attraction between spherical droplets of water phase in the W/O microemulsion. This is called percolation threshold (i.e., water droplets begin to contact with each other and network of channels are formed, which corresponds to the formation of a laminar structure that exhibits a gel-like appearance in the oil phase) [64]. Finally, by further increasing of water content above 50%, there was a decrease in the viscosity and continuous increase in the electrical conductivity (non-linear relationship) to higher values, which may be due to the presence of water as external phase and the formation of O/W microemulsion [65,66].

From these results, pseudo-ternary phase diagrams constructed using oleic acid as the oily phase, Cremophor EL as the surfactant, and PG as the co-surfactant in three different (S:C) weight ratios (1:1, 1:2, and 2:1, respectively) were selected to obtain microemulsion formulations. From each phase diagram, four formulations were selected from the microemulsion region for TA incorporation (0.05% *w/w*) and then were subjected to a thermodynamic stability study as described in Table 2.

### 3.4. Thermodynamic Stability Study

It is clear from Table 2 that only six formulations passed the thermodynamic stability tests. These stable formulations have oil to surfactant ratio of 0.64 to 0.86 *w/w*. Below and above this range, the formed microemulsions were found to lose their stability. Additionally, when microemulsion formulations were stored at low temperature (4 °C and −21 °C), as in the case of heating−cooling cycles and freeze−thaw cycles, they demonstrated reversible turbidity that easily disappeared by restoring at room temperature. In general, there was an optimum oil to surfactant ratio to obtain stable microemulsion. High surfactant concentrations may result in increasing emulsification and penetration of water into oil droplets, which leads to release of the oil into the aqueous phase [67]. In contrast, at high oil content, the surfactant will be insufficient for successful emulsification [68]. Those formulations that passed the thermodynamic stability tests were used for further characterization.

### 3.5. Microemulsion Characterization

The thermodynamically stable formulations were subjected to characterization according to pH, viscosity, conductivity, mean droplet size, PDI, and zeta potential and the results are presented in Table 6.

Regarding pH measurement, all prepared microemulsion formulations were found to have pH values ranged from 5.12 ± 0.2 to 5.89 ± 0.2, which are in the acceptable and tolerable pH range of the eye (i.e., 3.5–8.5) and are expected to cause no irritation. The ideal pH for optimum comfort when an ophthalmic preparation is instilled into the eye should be close to the pH of the tears (7.4). However, different pH values can be tolerated if it is buffered at low buffer capacity to enable the tears with their limited buffering capacity to adjust the pH to the physiological levels upon administration [35].

Another important character that should be assessed is the viscosity of the ophthalmic preparations, which affects the ocular contact time and thus the drainage of the drug by tears. It is clear from Table 6 that all formulations were found to exhibit viscosity values higher than the acceptable value of ophthalmic preparations (20 cP) [69], which were anticipated to increase the formulation residence time and hence its ocular bioavailability with keeping the advantage of reducing the instillation frequency per day [35]. The measured viscosities of the selected formulations were found to be affected by the water content, as water reduces the interaction between hydrophilic headgroups of the surfactant and thus decreases the viscosity [70]. Formulation F8, which possessed the highest water content (i.e., 60%), showed the lowest viscosity value (53 ± 1 cP). Additionally, there was a direct relationship between the surfactant to co-surfactant weight ratio and the formulation viscosity, which may be due to the change of the nature and shape of the internal phase [63]. Although F10 and F3 contained the same oil and water content, F10 exhibited significantly higher viscosity value (600 ± 4 cP) compared with that of F3 (293 ± 2 cP) (*p* < 0.05), which can be attributed to its higher surfactant to co-surfactant ratio [35]. 

The electrical conductivity could be considered as a useful tool to assess microemulsion structure based on the close relationship between the structure type (e.g., oil-continuous or water-continuous) and the microemulsion electro-conductive behavior [17]. High conductivity values were exhibited from water continuous formulations due to the presence of electroconductive channels [20]. As shown in Table 6, electrical conductivity values of the prepared formulations were found to range from 15 to 37 µs except F1 and F8. Formulation F1, which had a lower water content, showed lower conductivity value compared to F8, which had higher water content. Additionally, formulations with the same water and oil content (F3 and F10) showed the same conductivity values. The electrical conductivity values indicated that the prepared formulations were in the form of O/W phase system, which could be considered as an appropriate system for ophthalmic applications [71].

According to Table 6, all formulations possessed a droplet size in the range of 184–267 nm. Compared to F5 and F10, smaller mean droplet sizes were observed for other formulations, which can be attributed to their corresponding intermediate surfactant to co-surfactant concentration [72]. Formulation F5 showed large mean droplet size due to its high co-surfactant content, which in turn affects the property of the surfactant curvature (i.e., addition of co-surfactant causes the film to expand) [57]. However, the increased droplet size of F10 with low co-surfactant content can be attributed to the formation of a highly viscous liquid crystalline phase that increases the difficulty of spontaneous breakup of the oil–water interface [73]. Regarding PDI, all formulations (except F5) were found to exhibit PDI values below 0.5, which indicates narrow distribution of mean droplet size [25]. The PDI is also affected by the surfactant and co-surfactant concentrations, as the PDI increased by increasing the co-surfactant concentration. Therefore, small droplet size formation require an optimal surfactant and co-surfactant concentration, which must be defined for each surfactant, oil, and water combination [61]. Finally, all tested formulations showed negative zeta potential values ranged from −21.5 ± 2.3 to −27.1 ± 3.5 mV with a nonsignificant difference between them (except F5 which has −15 ± 1.4 mV). High zeta potential values have a role in microemulsion stabilization because the presence of high electrical charge in the system will cause repulsion between droplets leading to resistance of aggregation due to electrical stabilization [37,71]. 

### 3.6. In Vitro Release Experiments

**Figure 4:**
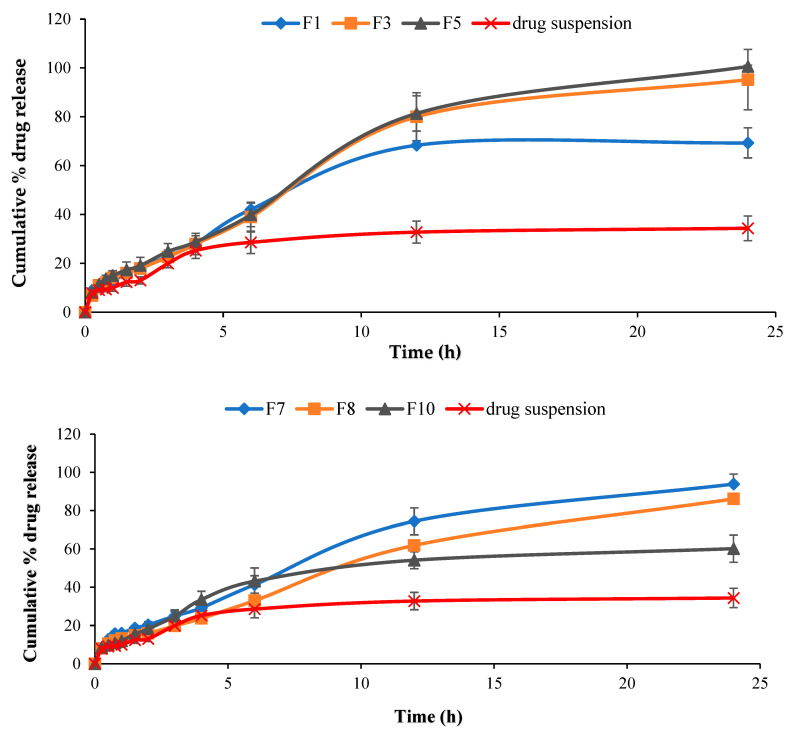
In vitro release pattern of triamcinolone acetonide from selected microemulsion formulations compared to its suspension in simulated tear fluid (pH 7.4). Data were expressed as mean value ± SD (*n* = 3).

The release patterns of the prepared microemulsion formulations after 24 h in comparison with TA suspension are demonstrated in Figure 4. From this figure, it is clear that drug release from various microemulsion formulations in simulated tear fluid was considerably more than that from free drug suspension (*p* < 0.05) which can be explained by faster release of the dissolved drug from small sized droplets which have large surface area [30]. 

Additionally, formulations F1 and F10 showed significantly slower drug release rate after 24 h than that from other tested formulations (*p* < 0.05) which can be correlated to their physicochemical characteristics. It appears that drug liberation from the tested formulations is influenced by their rheological behavior. The cumulative amount of drug released after 24 h was significantly high from the formulations with lower viscosity, which assisted drug diffusion. In contrast, F1 and F10 were found to exhibit slower drug release which may be due to their corresponding higher viscosity. This can be explained by Vlaia et al. [29], who stated that by increasing microemulsion viscosity, the stability of the formed micelles was enhanced and resulted in decreasing flux of the drug [59].

The drug release from various formulations was found to follow Higuchi diffusion model which give the best fit suggesting diffusion as the main release mechanism (Table S1, Supplementary Data). The calculated exponent (*n*) value of this model for most formulations was found to be ≤0.5 suggesting that, the release of TA from these formulations is mediated by Fickian diffusion.

### 3.7. Further Characterization of the Selected Formulation

Formulation F3 was chosen as optimized formulation due to its small droplet size (i.e., 211.9 ± 1.4 nm), narrow PDI (i.e., 0.217), high negative zeta potential value (i.e., −25.7 ± 1.7), acceptable pH value (i.e., 5.39), and high corresponding cumulative % drug release after 24 h (i.e., 95% ± 8). Thus, F3 was selected for further evaluation according to rheological behavior, morphological examination by transmission electron microscope, and long-term stability study.

#### 3.7.1. Rheological Behavior

According to Figure S3 (Supplementary Data), F3 exhibited pseudoplastic flow as shown by shear thinning and decreasing in the viscosity with increasing angular velocity. Ophthalmic formulations with this type of flow are preferred because the formulation is viscous to promote ocular retention while display less resistance during blinking without causing patient discomfort [21]

#### 3.7.2. TEM

**Figure 5:**
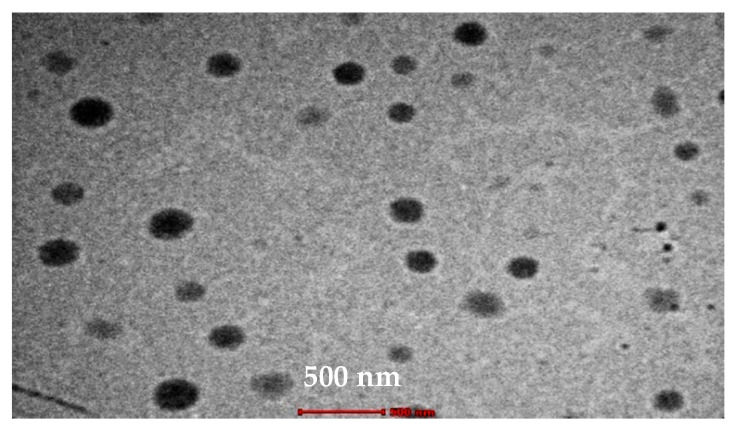
TEM photograph of the selected microemulsion formed (F3) (×26,000). Size = around 242 nm.

As shown in Figure 5, all droplets exhibit a spherical shape with no aggregation signs. The droplet size is in accord with the results obtained from droplet size analysis using zeta sizer.

#### 3.7.3. Stability Study

According to the stability results presented in Table 7, at room temperature (25 °C), the selected formulation (F3) showed nonsignificant changes in its appearance, droplet size, zeta potential or pH value when stored for six months. In contrast, at 4 °C, the selected formulation (F3) showed turbid appearance and significant changes (*p* < 0.05) in both droplet size and zeta potential after two months. This instability may be due to internal phase coagulation [26]. As a recommendation from this study, the selected formulation (F3) should be stored at room temperature to maintain its long-term stability.

### 3.8. In Vivo Studies

In vivo studies are important to assess any observable damage and histopathological changes of the ocular tissues caused by the selected microemulsion formulation and to evaluate its performance in the treatment of uveitis by using uveitis induced rabbit eye model. 

#### 3.8.1. Ocular Irritation Test

The results of the ocular irritation test (as shown in Figures S4 and S5 (Supplementary Data)) revealed that treatment with non-medicated F3 did not cause any damage to the corneal surface or any signs of ocular irritation such as redness, tearing, or swelling. In addition, histological examination showed no evidence of pathological changes in the cornea, iris, ciliary body and its processes. This indicates that the selected microemulsion formulation is non-irritant which may be due to the acceptable percentage of surfactant and co-surfactant (i.e., 35%) and tolerability of oleic acid used in the selected microemulsion (F3) preparation. According to information provided by the manufacturer (BASF), the instillation of 30% aqueous solutions of Cremophor EL had no irritant effect on rabbits’ eyes; however, 50% aqueous solution caused slight irritation with lacrimation, which disappeared rapidly [17]. Additionally, solutions of up to 50% PG caused no irritations to the rabbit eye, whereas the undiluted application was associated with a slight conjunctival redness [35].

#### 3.8.2. Induction of Uveitis

Bovine serum albumin was tested for uveitis induction at two dose strengths (i.e., 10 mg and 40 mg). It was found that intravitreal injection of 10 mg BSA resulted in few uveitis symptoms and subsided rabidly, while intravitreal injection of 40 mg resulted in observable symptoms that lasted for long time. The experimental uveitis produced by this technique was characterized by a marked dilatation of the blood vessels in the iris and ciliary body region, a prolonged flare due to the liberation of large quantities of protein, and the appearance of white blood cells in the anterior chamber. These observations are in agreement with Kulkarni et al. [41].

##### Clinical Observations of Uveitis Symptoms

**Figure 6:**
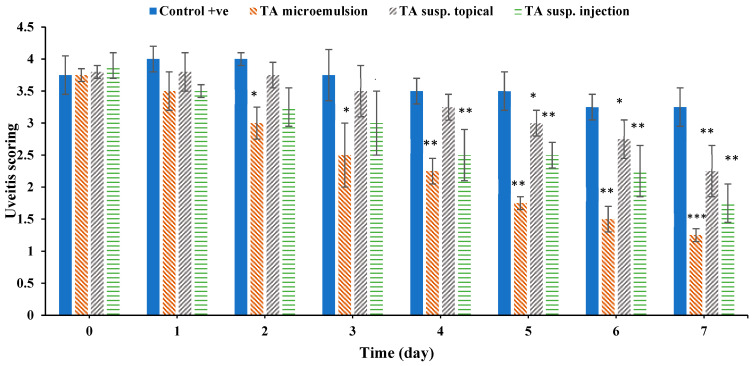
Clinical uveitis score of the tested groups during seven days treatment. Data were expressed as mean value ± SD (*n* = 3). * *p* < 0.05, ** *p* < 0.01, *** *p* < 0.001, significant reduction of inflammation compared to untreated positive control group with given zero inflammatory score for the negative control group.

Figure S6 (Supplementary Data) demonstrates the clinical signs of uveitis in all tested groups at three different occasions: before treatment, after four days of treatment, and finally after seven days of treatment. Figure 6 illustrates the inflammation scores of the uveitis-induced symptoms during seven days of treatment with the selected TA microemulsion F3 (Group II), topical TA suspension (Group III), and subconjunctival injected TA suspension (Group IV) compared to positive control (Group I) by considering zero inflammatory score for the negative control group. 

After 24 h from starting the treatment, all treated groups as well as the positive control group demonstrated a similar degree of inflammation. After two days of treatment, F3 and TA injection showed more significant reduction of the inflammation scores (*p* < 0.05) compared with TA suspension and positive control group. At the end of the treatment, it was observed that the three groups treated with the drug suspension, injection, or microemulsion (F3) showed a significant reduction in the inflammation compared to positive control group (*p* < 0.05). However, by comparing the effect of the three dosage forms, it was found that TA microemulsion formulation (F3) had the most significant control of eye inflammation (*p* < 0.05) followed by TA injection then TA suspension. The more pronounced efficacy of the microemulsion formulation can be attributed to the greater availability of the solubilized form of the drug from their nano-sized droplets with large surface area which permits faster absorption and easier penetration of the drug facilitating its delivery more deeply reaching the posterior segments of the eye [14]. 

##### Anterior Chamber WBCs Count and Protein Content

**Figure 7:**
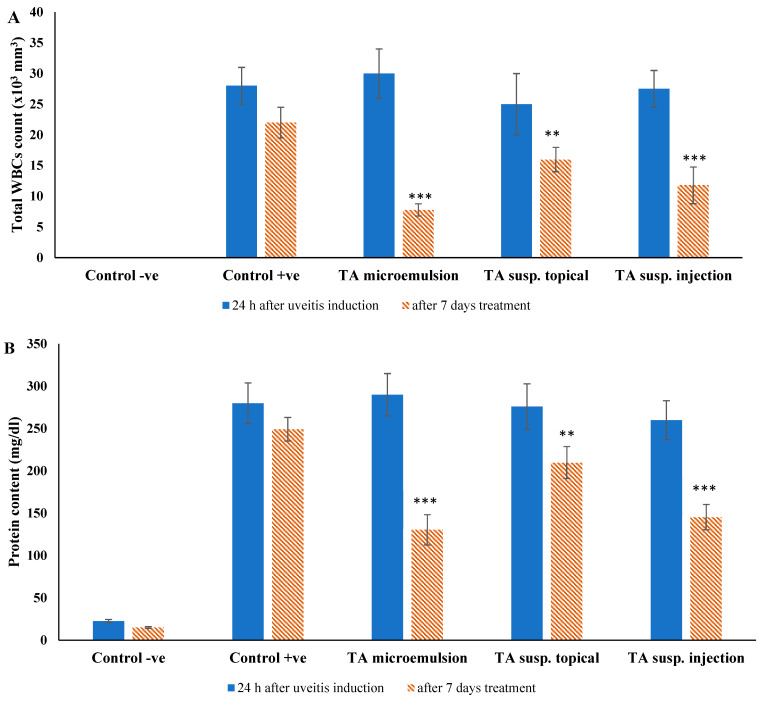
Aqueous humor analysis, (**A**) white blood cell (WBC) count, and (**B**) protein content after seven days treatment in tested groups. Data were expressed as mean value ± SD (*n* = 3). ** *p* < 0.01, *** *p* < 0.001 significantly differ from initial values obtained 24 h after induction of uveitis with given zero white blood cells count for the negative control group.

Figure 7 represents the number of WBCs and the protein content in the aqueous humor of the animal groups after seven days of treatment with TA microemulsion F3, topical TA suspension, and subconjunctival injected TA suspension compared to the initial values after uveitis induction. Before induction of uveitis, the aqueous humor did not contain any WBCs but had an average protein content of 22 ± 2 mg/dL. Twenty-four hours after BSA injection (uveitis induction), intense inflammation was observed in the anterior chamber of the tested eyes accompanied with marked elevation of both WBCs and protein content, which indicated the disruption of blood-aqueous barrier (BAB) integrity.

TA microemulsion (F3) reduced the inflammatory cell count and protein content by 74% and 55%, respectively, topical TA suspension downgraded inflammatory cells by 36% and protein content by 24%, and finally, subconjunctival injected TA suspension decreased inflammatory cells by 57% and protein content by 44%. The positive control group showed a reduced WBC count and protein content by 7% and 11%, respectively. From the previous results, all tested formulations caused a significant reduction (*p* < 0.05) of WBC count and protein content in the aqueous humor compared with positive control group. Further, the TA microemulsion (F3)-treated group showed the most significant reduction (*p* < 0.05) in WBCs and protein content compared with topical TA suspension subconjunctival injected TA suspension-treated groups. These results confirm the superior therapeutic efficacy of TA in the treatment of uveitis when formulated in a microemulsion dosage form compared to other dosage forms. 

##### Histopathology

**Figure 8:**
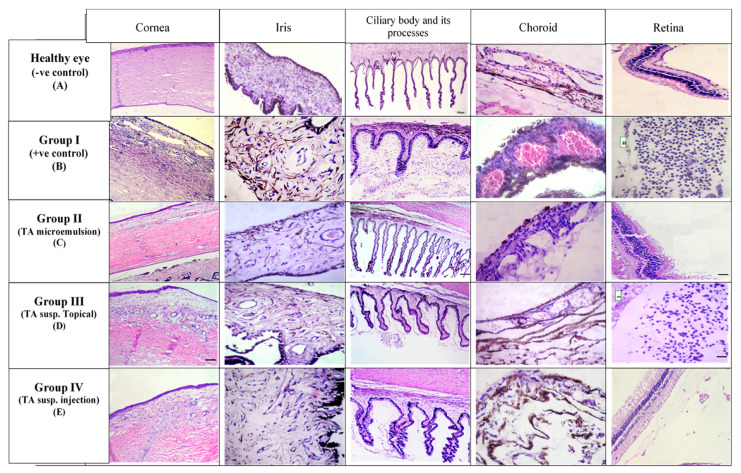
Histopathological examination of different sectors of the rabbits’ eye (cornea, iris, ciliary body and its processes, choroid, and retina) after their treatment with triamcinolone acetonide (TA) microemulsion F3 (**Group II**), topical TA suspension (**Group III**), and subconjunctival injection TA suspension (**Group IV**) compared with negative control (healthy eye) and positive control (**Group I**).

Figure 8 shows the histopathological photographs of different sectors of rabbits’ eyes after treatment with TA microemulsion F3 (group II), topical TA suspension (group III), and subconjunctival injected TA suspension (group IV) compared with negative and positive control groups.

Different photographs of the negative control group represented the normal layers of different sectors of healthy eye including (cornea, iris, ciliary body and its processes, choroid, and retinal tissues). In Group II, treated with the selected TA microemulsion formulation (F3), a marked decrease in the intensity of corneal inflammatory cellular reaction was observed and scored (+2). There was no evidence of inflammatory cellular reaction in the ciliary body (+1) with less or no edema of the iris stroma (+1). Moreover, there was less hyperemia of the choroidal blood vessels (+2) with no evidence of retinal inflammatory cellular reaction.

In Group III, topically treated with TA suspension, corneal inflammatory cellular reaction with intracellular edema scored (+3) was observed. Iris intracellular edema (+2) and inflammatory cellular reaction of the ciliary body (+2) were also observed. Moreover, hyperemia of the choroidal blood vessels (+3) with retinal cellular reaction (+3) were also observed.

In Group IV, received a single subconjunctival injection of TA suspension, corneal inflammatory cellular reaction with intracellular edema scored (+2) was observed. Inflammatory cellular reaction of the ciliary body (+1) with iris intracellular edema (+2) were observed. Finally, less hyperemia of the choroidal blood vessels (+2) with less cellular reaction around the retina (+1) was observed.

**Figure 9:**
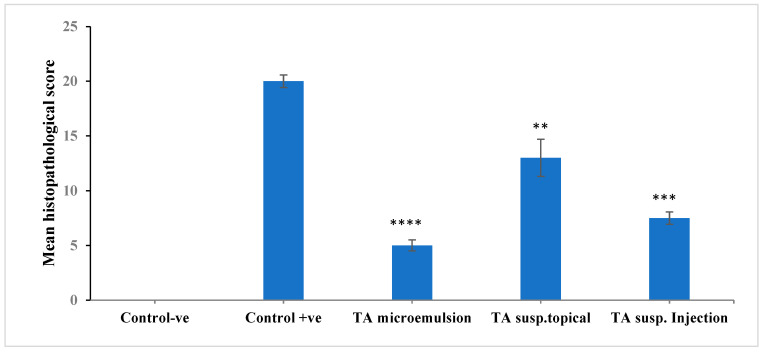
The mean histopathological scores of uveitis-induced rabbits after seven days treatment with TA microemulsion (F3), TA topical suspension, and subconjunctival injected TA suspension compared to untreated positive control group with calculated zero mean histopathological score for the negative control group. Data were expressed as mean value ± SD (*n* = 3). ** *p* < 0.01, *** *p* < 0.001 and **** *p* < 0.0001 highly significant reduction of inflammation compared to positive control group.

For comparison, the average means of the histopathological scores observed in all treated groups were detected and represented in Figure 9. It was noted that all treated groups caused a significant reduction (*p* < 0.05) in inflammatory reaction in ocular tissues compared to untreated group (Group I, positive control). Moreover, a significant difference was also observed in Group II, which received selected formulation F3 in comparison with Group III and Group IV. 

The results obtained from uveitis scoring, WBC count, protein content, and from histopathological findings approve the effectiveness of TA in inhibiting several parameters of inflammation in this immunogenic rabbit model. Additionally, the selected microemulsion formulation (F3) was significantly effective in reducing the inflammation (*p* < 0.05) compared to topical TA suspension and subconjunctival injected TA suspension.

The enhanced controlling effect of uveitis symptoms achieved by microemulsion formulation (F3) can be explained by the presence of the drug in solubilized form in the nano-sized microemulsion droplets with large surface area, which allows for faster absorption and easier penetration of the drug [74]. Another reason for enhancing the bioavailability of microemulsion formulation is the ability of their nanodroplets to be adsorbed on the corneal surface and avoiding elimination by lacrimal drainage and so they act as a reservoir of the drug [14]. Additionally, the high viscosity of the developed microemulsion compared to suspension helps to increase the retention of the formulation for prolonged time in contact with the cornea. Moreover, the nonionic surfactant (Cremophor EL) used in the formulation of microemulsion was reported to have an established bioactive penetration-enhancing effect by alteration of the membrane properties through disrupting tear film, mucin, and the integrity of the epithelia by loosening tight junctions [19]. Additionally, the used oil (oleic acid) was reported to increase permeation of the formulation through ocular tissues resulting in facilitating drug delivery to the posterior segment of the eye [22]. 

All these factors render microemulsion formulation more effective in uveitis treatment than topical (i.e., low ability to penetrate ocular tissues) or subconjunctival injection of the drug suspension (i.e., need to be injected several times).

## 4. Conclusions

In this study, different pseudo-ternary phase diagrams were constructed using water titration method. The impact of oil, surfactant, co-surfactant, and water content on microemulsion formation were investigated. Based on the microemulsion area, oleic acid, Cremophor EL, and PG were chosen as an oil, surfactant, and co-surfactant, respectively. TA was successfully incorporated into different stable microemulsion formulations with acceptable physicochemical properties. Regarding pH, droplet size, and viscosity, F3 was selected and considered to be acceptable to be applied to the eye. F3 was non-irritant and cause no damage to ocular tissues. The in vivo studies revealed that F3 was significantly reduced inflammation signs, protein content, and inflammatory cells in experimentally induced uveitis compared to TA suspension either applied topically or taken as subconjunctival injection. The results obtained in this study show the effectiveness of microemulsion for the ocular delivery of TA in the treatment of uveitis with high patient compliance and prevent complications associated with injection. 

## Figures and Tables

**Table 1:** Composition of the different pseudo-ternary phase diagrams.

Group	Oil Phase	Surfactant (S)	Co-Surfactant (C)	S:C (S_mix_)
I	Castor oil	Tween 80	PEG 400	1:1
		Brij 35		
		Cremophor EL		
II	Oleic acid	Tween 80	PEG 400	1:1
		Brij 35		
		Cremophor EL		
III	Oleic acid	Cremophor EL	PEG 400	1:1
				1:2
				2:1
IV	Oleic acid	Cremophor EL	Glycerol	1:1
				1:2
				2:1
V	Oleic acid	Cremophor EL	PG	1:1
				1:2
				2:1

**Table 2:** Composition of microemulsion formulations loaded with triamcinolone acetonide (0.05% *w/w*) and their thermodynamic stability results.

Code	Ratio (S:C)	Composition (% *w/w*)					Thermodynamic Stability Study
		Oleic Acid	Cremophor EL	PG	Phosphate Buffer	Oil/Surf. (*w/w*)	
F1	1:1	20	23.5	23.5	33	0.85	√
F2	1:1	10	20	20	50	0.5	-
F3	1:1	15	17.5	17.5	50	0.86	√
F4	1:1	8	16	16	60	0.5	-
F5	1:2	13	16.7	33.3	37	0.78	√
F6	1:2	5	15	30	50	0.33	-
F7	1:2	10	13.3	27.7	50	0.73	√
F8	1:2	8	10.7	21.3	60	0.76	√
F9	2:1	10	26.7	13.3	50	0.38	-
F10	2:1	15	23.3	11.7	50	0.64	√
F11	2:1	8	21.3	10.7	60	0.38	-
F12	2:1	20	20	10	50	1	-

**Table 3:** The scoring system used to assess the clinical condition of the eye [41].

Score	Evaluation
1	Mild vasodilatation of the iris and conjunctiva
2	Moderate vasodilatation of the iris and conjunctiva
3	Moderate vasodilatation of the iris and conjunctiva and slight flare
4	Sever vasodilatation of the iris and conjunctiva with heavy flare and fibrin strands

**Table 4:** Saturation solubility of triamcinolone acetonide in various vehicles at 37 ± 0.5 °C.

Vehicle	Solubility (µg/mL) ^≠^
Oleic acid	1500 ± 50
Castor oil	3120 ± 80 *
Olive oil	100 ± 5
Corn oil	75 ± 10
Soya bean oil	95 ± 8
Tween 80	4700 ± 300
Cremophor EL	4460 ± 309
Brij 35	8540 ± 110 **
PEG 400	540 ± 13 ***
PG	390 ± 10
Glycerol	150 ± 15

^≠^ Data were expressed as mean value ± SD (*n* = 3). * Significantly higher than other oils (*p* ˂ 0.05). ** Significantly higher than other surfactants (*p* ˂ 0.05). *** Significantly higher than other co-surfactants (*p* ˂ 0.05).

**Table 5:** Microemulsion area percentage related to pseudo-ternary phase diagrams composed of three different co-surfactants (PEG 400, g/lycerol, and PG) with oleic acid (oil) and Cremophor EL (surfactant), maximum solubilized oil and phase transition.

Co-Surfactant	S:C Ratio	% Microemulsion Area	Maximum Solubilized Oil (%*w/w*)	% A_fluid_	% A_bi_
PEG 400	1:1	15	8	13	2
	1:2	11	5	8.5	2.5
	2:1	29	15	14	15
Glycerol	1:1	12.5	23	9	3.5
	1:2	9	18	7	2
	2:1	18	33	3	15
PG	1:1	24.6	33	19.7	4.9
	1:2	12	16	12	-
	2:1	32.4	33	16.8	15.6

A_fluid_: area of fluid (O/W) and (W/O), A_bi_: area of bicontinuous.

**Table 6:** Physicochemical characterization of the selected microemulsion formulations in the presence of triamcinolone acetonide.

Code	pH ^#^	Viscosity (cP) ^#^	Electrical Conductivity (µS) ^#^	Mean Droplet Size (nm) ^#^	PDI	Zeta Potential (mV) ^#^
F1	5.12 ± 0.2	300 ± 1.5	6 ± 0.3	201.4 ± 6.3	0.44	−21.5 ± 2.3
F3	5.39 ± 0.2	293 ± 2	15 ± 0.5	211.9 ± 1.4	0.217	−25.7 ± 1.7
F5	5.4 ± 0.2	146 ± 1	37 ± 0.2	250.1 ± 11.3 *	0.769	−15.3 ± 1.4 *
F7	5.63 ± 0.2	126 ± 2	34 ± 0.4	188.7 ± 3.7	0.448	−23.1 ± 2
F8	5.89 ± 0.2	53 ± 1	60 ± 0.7	184.3 ± 1.9	0.408	−27.1 ± 3.5
F10	5.45 ± 0.2	600 ± 4	15 ± 0.2	267.05 ± 6.9 *	0.541	−21.2 ± 1.6

**^#^** Data were expressed as mean value ± SD (*n* = 3). * Significantly differ from other formulations (*p* ˂ 0.05).

**Table 7:** Evaluation data of the stability study of the selected microemulsion formulation (F3) at room temperature (25 °C) and refrigeration temperature (4 °C).

Parameters ^≠^	Temp.	Time (month)						
		0	1	2	3	4	5	6
**Droplet size** **(nm)**	25 °C	220 ± 3.8	226 ± 3.7	231 ± 5.9	224 ± 3.5	196 ± 1.6	210 ± 1.5	224 ± 2
	4 °C	220 ± 3.8	232 ± 2.6	250 * ± 3.4	283 ** ± 2	290 ** ± 1.5	297 ** ± 2.5	310 ** ± 3
**PDI**	25 °C	0.22 ± 0.02	0.3 ± 0.01	0.32 ± 0.02	0.35 ± 0.04	0.33 ± 0.05	0.37 ± 0.03	0.4 ± 0.02
	4 °C	0.22 ± 0.02	0.35 ± 0.04	0.412 ± 0.02	0.52 ± 0.03	0.51 ± 0.03	0.55 ± 0.01	0.5 ± 0.03
**Zeta potential (mV)**	25 °C	−25 ± 1.1	−24.5 ± 2.5	−23.1 ± 1.5	−22.3 ± 2	−22 ± 2.6	−21.5 ± 1	−21 ± 2
	4 °C	−25 ± 1.1	−24.06 ± 2	−21.3 ± 1.5	−19.29 ± 3	−18 ± 2.2	18.5 ± 2.5	17.9 ± 1.6
**pH**	25 °C	5.39 ± 0.02	5.46 ± 0.04	5.5 ± 0.05	5.6 ± 0.04	5.75 ± 0.03	5.81 ± 0.02	5.9 ± 0.02
	4 °C	5.39 ± 0.03	5.45 ± 0.05	5.55 ± 0.07	5.6 ± 0.04	5.71 ± 0.02	5.77 ± 0.03	5.8 ± 0.02
**Appearance**	25 °C	Translucent	Translucent	Translucent	Translucent	Translucent	Translucent	Translucent
	4 °C	Translucent	Translucent	Slight turbidity	Slight turbidity	Turbid	Turbid	Turbid

^≠^ Data were expressed as mean value ± SD (*n* = 3). * Significantly differ from initial value (*p* < 0.05); ** highly significant difference from initial value (*p* ˂ 0.01).

## Data Availability

Not applicable.

## Supplementary Materials

The following are available online at https://www.mdpi.com/1999-4923/13/4/444/s1, Figure S1: Spectrophotometric scanning of triamcinolone acetonide in methanol. Figure S2. Calibration curve of triamcinolone acetonide in methanol. Figure S3. Calibration curve of triamcinolone acetonide in simulated tear fluid at pH 7.4. Figure S4. Pseudo-ternary phase diagrams of Group I containing castor oil as oily phase, (a)Tween 80, (b) Brij 35, and (c) Cremophor EL as surfactants and PEG 400 as a co-surfactant in ratio S:C (1:1) and their corresponding microemulsion areas as a percentage related to total area of the pseudo-ternary diagram. Figure S5. Pseudo-ternary phase diagrams of Group II containing oleic acid as oily phase, (a) Tween 80, (b) Brij 35, and (c) Cremophor EL as surfactants and PEG 400 as a co-Surfactant in ratio S:C (1:1) and their corresponding microemulsion areas as a percentage related to total area of the pseudo-ternary diagram. Figure S6. Rheology profile of the selected formulation F3. Data were expressed as mean value ± SD (*n* = 3). Figure S7. Photographs of rabbit’s eye after seven days topical instillation of (A) microemulsion formulation (F3) and (B) control eye. Figure S8. Histological sections of rabbit’s eye at the end of Draize test after seven days instillation of (A) microemulsion formulation (F3) (B) control eye stained by H and E, examined using light microscope x100. Figure S9. Representative photographs of clinical examination of the eyes of uveitis-induced rabbits. Group I (control + ve), Group II (F3), Group III (triamcinolone acetonide suspension) and Group IV (triamcinolone acetonide injection) at three different occasions: before treatment, after four days of treatment, and after seven days of treatment. Figure S1. Spectrophotometric scanning of triamcinolone acetonide in methanol. Figure S2. Calibration curve of triamcinolone acetonide in methanol. Figure S3. Calibration curve of triamcinolone acetonide in simulated tear fluid at pH 7.4. Table S1. Release kinetics of the selected microemulsion formulations.
