# Supplementary material for: Development of Triamcinolone Acetonide-Loaded Microemulsion as a Prospective Ophthalmic Delivery System for Treatment of Uveitis: In Vitro and In Vivo Evaluation
Source: Pharmaceutics. 2021 Mar 25;13(4):444. doi: 10.3390/pharmaceutics13040444 (PMC8064451; doi:10.3390/pharmaceutics13040444)
Supplement: Supplementary file 1 [file pharmaceutics-13-00444-s001.pdf]

pharmaceutics

Supplementary Materials: Development of Triamcinolone Acetonide-Loaded Microemulsion as a Prospective Ophthalmic Delivery System for Treatment of Uveitis: In Vitro and In Vivo Evaluation

Alaa Mahran, Sayed Ismail and Ayat A. Allam *

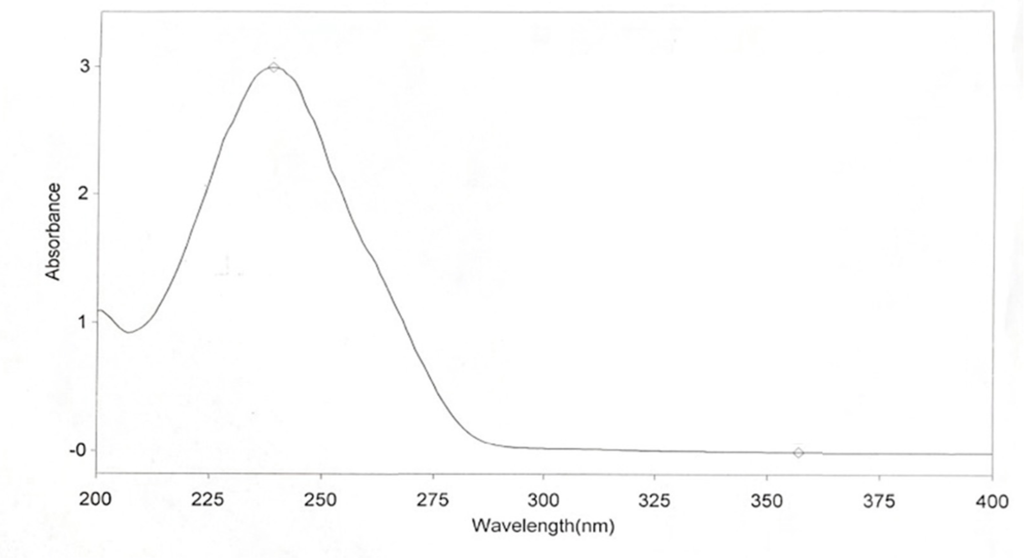

Figure S1. Spectrophotometric scanning of triamcinolone acetonide in methanol.

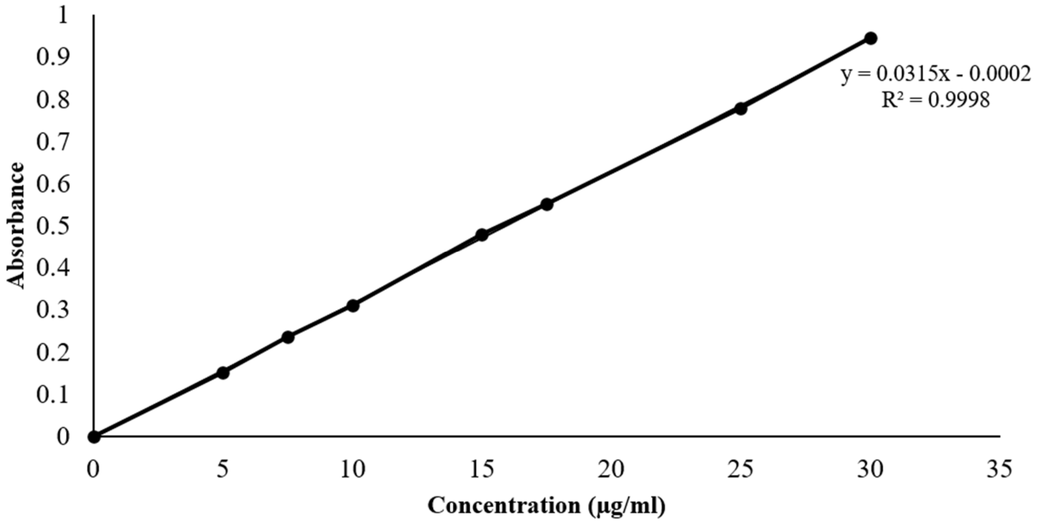

Figure S2. Calibration curve of triamcinolone acetonide in methanol.

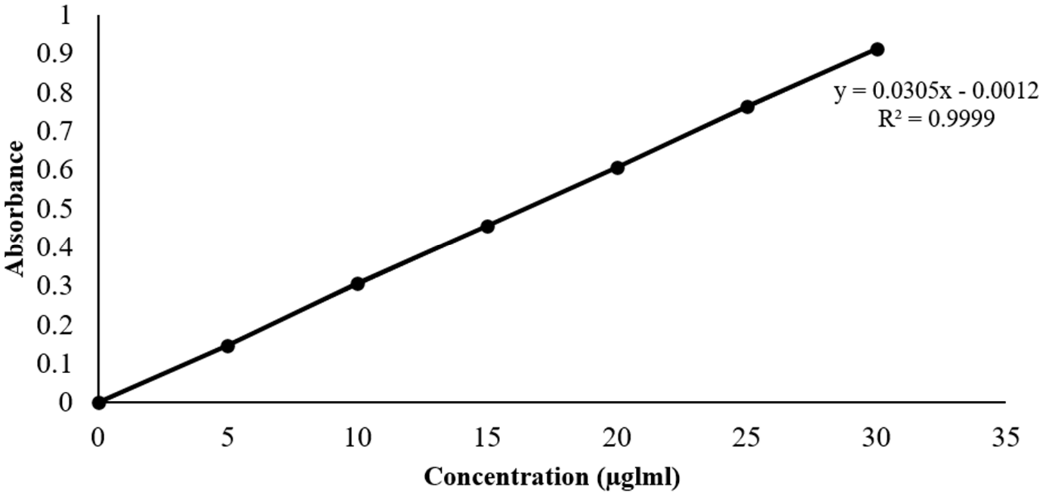

Figure S3. Calibration curve of triamcinolone acetonide in simulated tear fluid at pH 7.4.

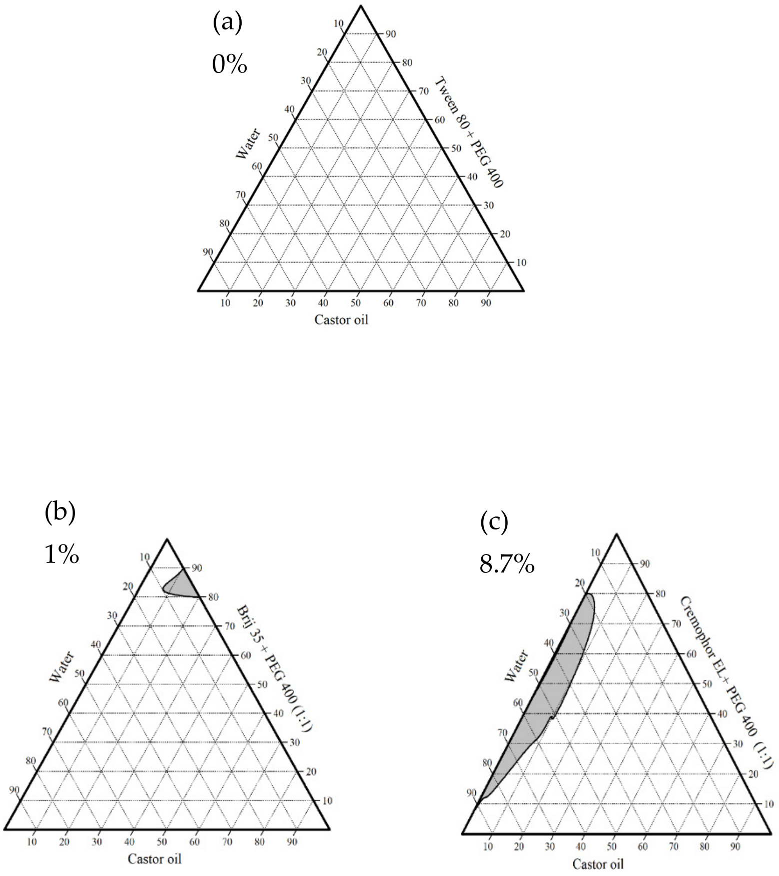

Figure S4. Pseudo-ternary phase diagrams of group I containing castor oil as oily phase, (a)Tween 80, (b) Brij 35 and (c) Cremophor EL as surfactants and PEG 400 as a co-surfactant in ratio S:C (1:1) and their corresponding microemulsion areas as a percentage related to total area of the pseudo-ternary diagram.

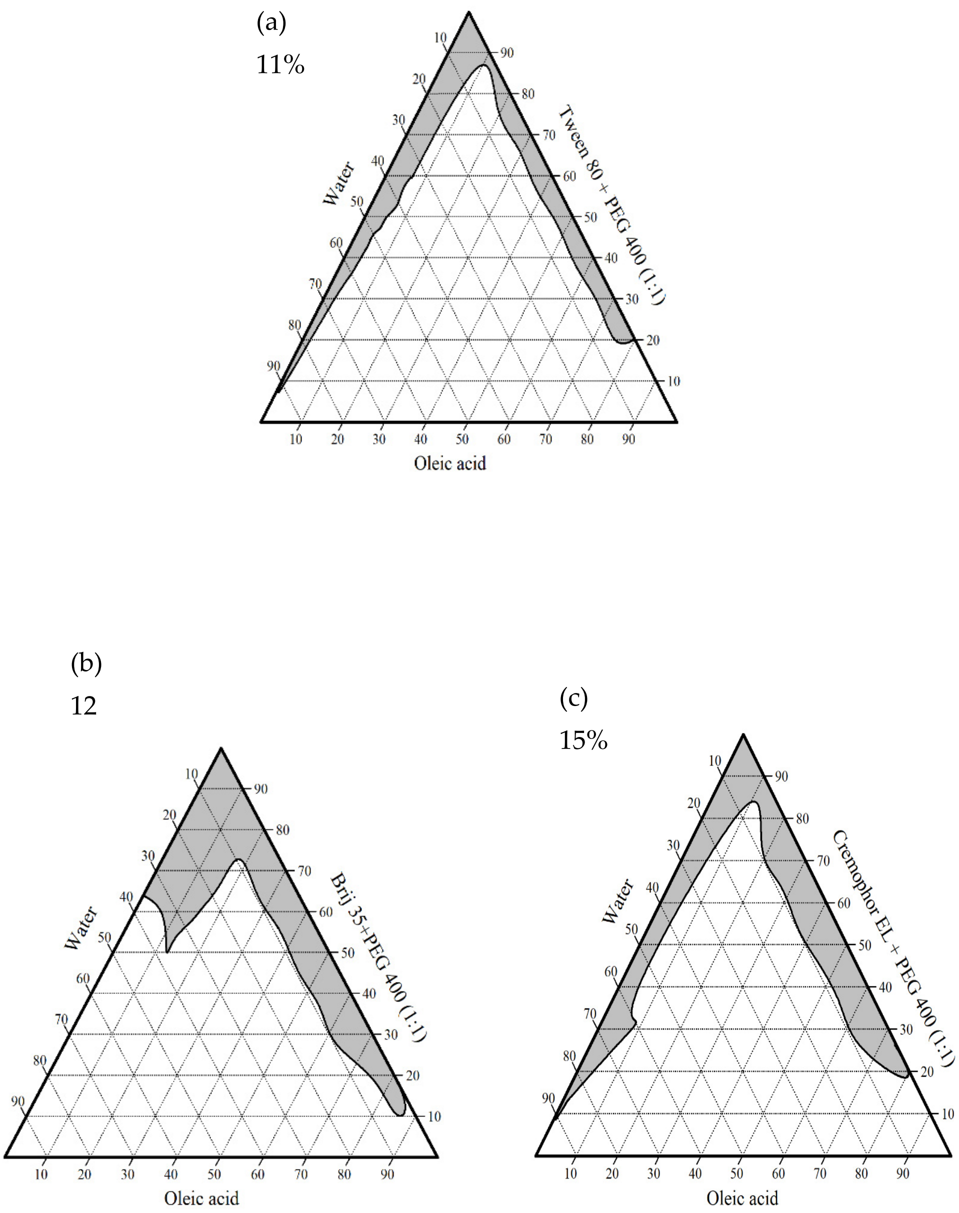

Figure S5. Pseudo-ternary phase diagrams of group II containing oleic acid as oily phase, (a) Tween 80, (b) Brij 35 and (c) Cremophor EL as surfactants and PEG 400 as a co-Surfactant in ratio S:C (1:1) and their corresponding microemulsion areas as a percentage related to total area of the pseudo-ternary diagram.

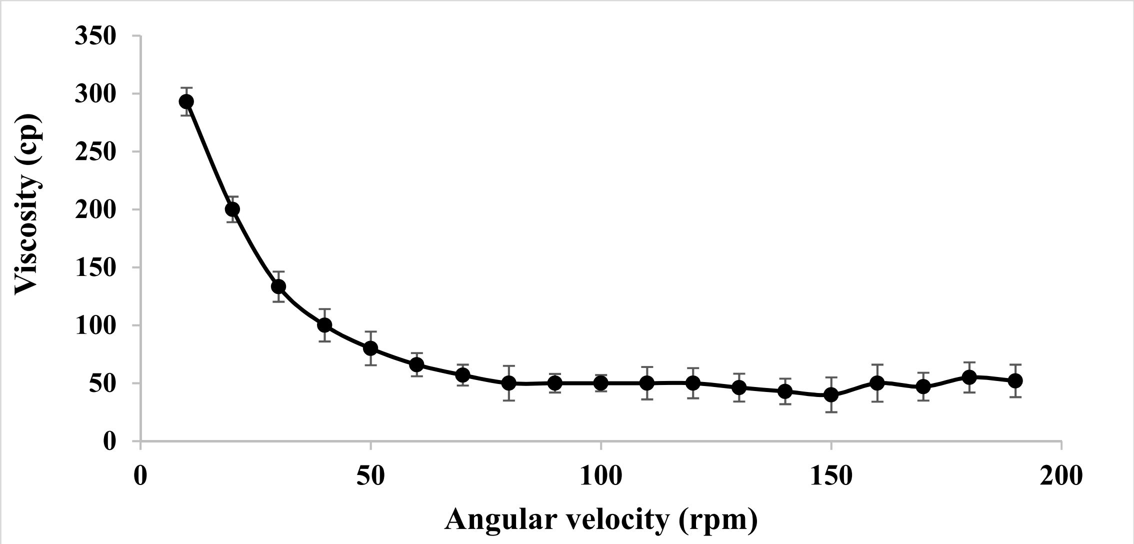

Figure S6. Rheology profile of the selected formulation F3. Data was expressed as mean value ± SD (*n* = 3).

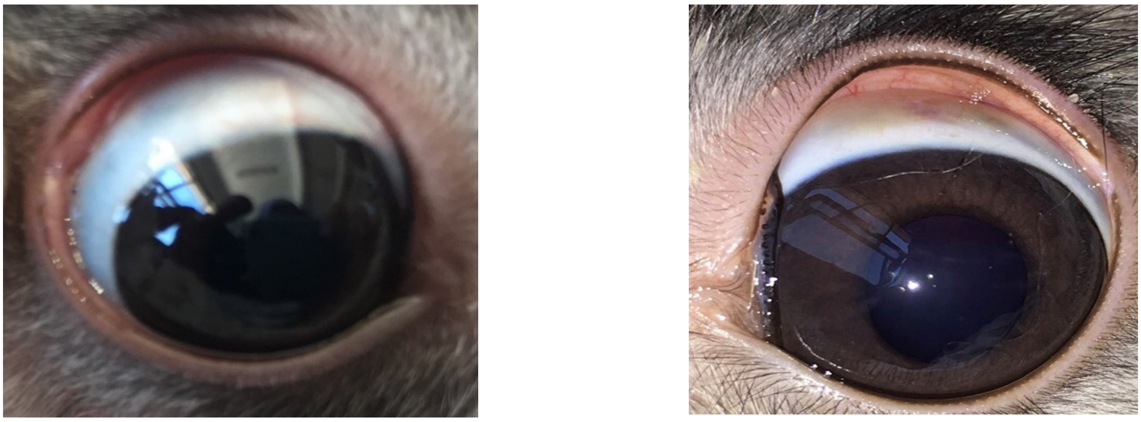

(a) (b)

Figure S7. Photographs of rabbit's eye after 7 days topical instillation of (a) microemulsion formulation (F3) and (b) control eye.

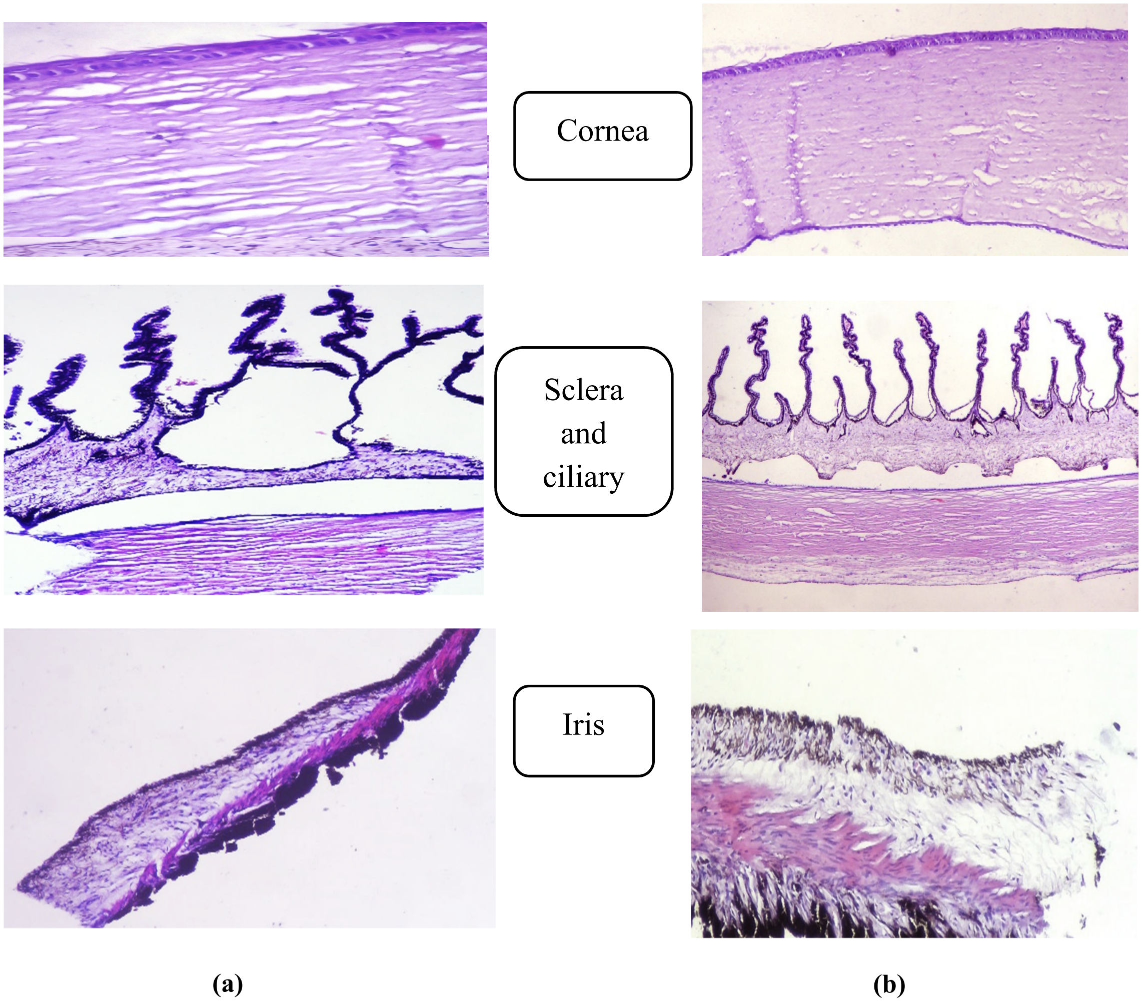

Figure S8. Histological sections of rabbit's eye at the end of Draize test after 7 days instillation of (a) microemulsion formulation (F3) (b) control eye stained by H&E, examined using light microscope x100.

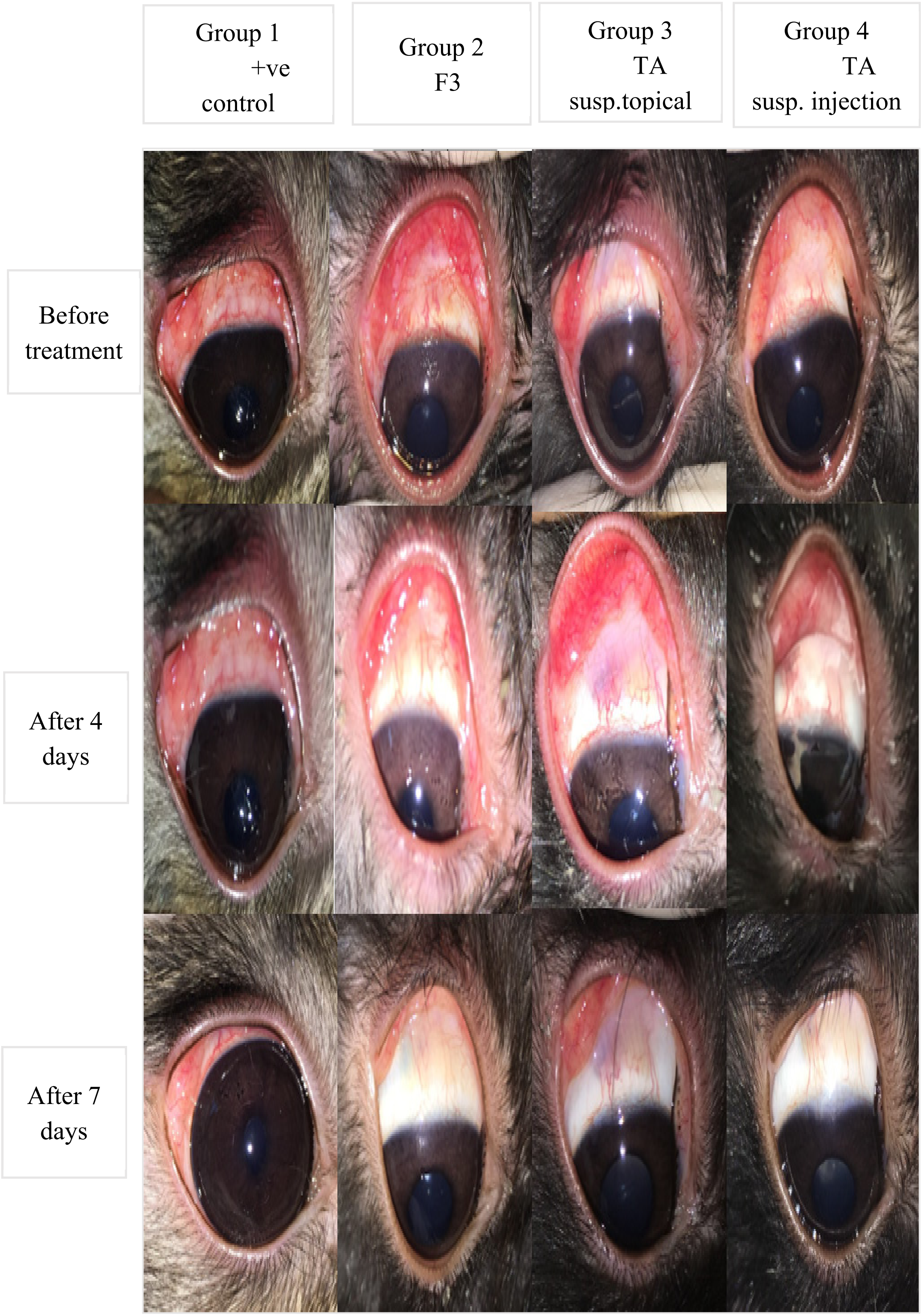

Figure S9. Representative photographs of clinical examination of the eyes of uveitis induced rabbits. Group I (control +ve), group II (F3), group III (triamcinolone acetonide suspension) and group IV (triamcinolone acetonide injection) at three different occasions: before treatment, after 4 days of treatment and after 7 days of treatment.

Table S1. Release kinetics of the selected microemulsion formulations.

Formulation	Zero Order (r)*	First Order (r)*	Higuchi Diffusion (r)*	Korsmeyer-Peppas (n)**
F1	0.91	0.078	**0.97**	0.47
F3	0.96	0.42	**0.98**	0.50
F5	0.96	0.59	**0.98**	0.47
F7	0.96	0.36	**0.98**	0.45
F8	0.98	0.21	**0.98**	0.48
F10	0.88	-0.01	**0.97**	0.50

* r = correlation coefficient; ** n = release exponent.

Different equations of the release kinetic models:

$$\text{zero order : } (\boldsymbol{m_0 = m_t - K_0 t}) \quad (1)$$

$$\text{first order model: } \left(\mathbf{Log\ m_t = Log\ m_0 - \frac{K_1 t}{2.303}}\right) \quad (2)$$

$$\text{Higuchi diffusion model: } (\boldsymbol{m_0 - m_t = K_h t^{1/2}}) \quad (3)$$

$$\text{Korsmeyer-Peppas equation: } \left(\boldsymbol{\frac{M_t}{M_\infty} = K\ t^n}\right) \quad (4)$$

where m_0 is the initial drug amount in the formulation, m_t is the remaining drug amount in the formulation at time (t), t is time (h) and K is the rate constant of the specified order. The regression coefficient values (r^2) were calculated for these models.
